# Supplementary figures and images for: Mitotic replisome disassembly depends on TRAIP ubiquitin ligase activity
Source: Life Sci Alliance. 2019 Apr 12;2(2):e201900390. doi: 10.26508/lsa.201900390 (PMC6464043; doi:10.26508/lsa.201900390)

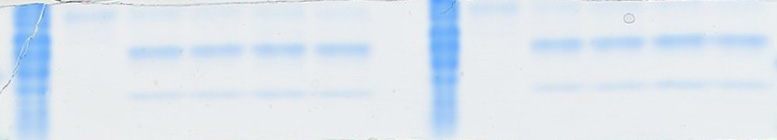

Supplement: Supplementary file 1 [file LSA-2019-00390_SdataF1.jpg]

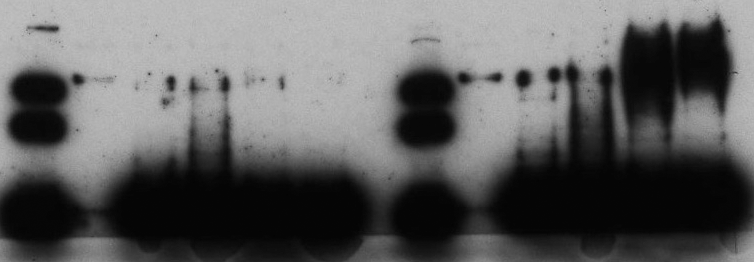

Supplement: Supplementary file 2 [file LSA-2019-00390_SdataF1.1.tif]

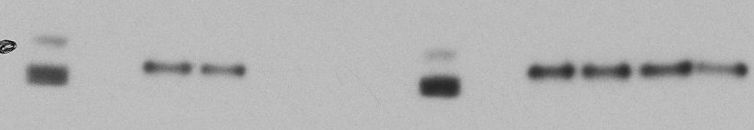

Supplement: Supplementary file 3 [file LSA-2019-00390_SdataF1.2.tif]

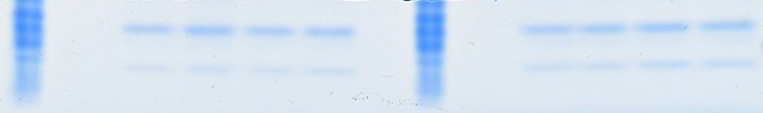

Supplement: Supplementary file 4 [file LSA-2019-00390_SdataFS2.jpg]

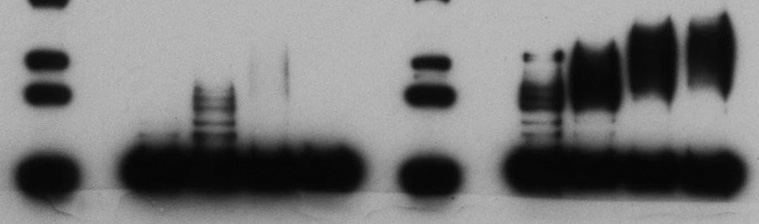

Supplement: Supplementary file 5 [file LSA-2019-00390_SdataFS2.1.jpg]

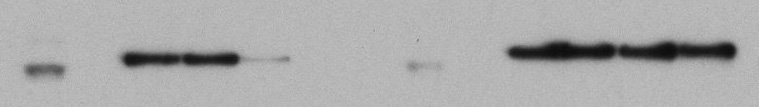

Supplement: Supplementary file 6 [file LSA-2019-00390_SdataFS2.2.jpg]
